# Human TERT Promoter Mutations in Atypical and Anaplastic Meningiomas

**DOI:** 10.3390/diagnostics11091624

**Published:** 2021-09-06

**Authors:** Marta Mellai, Omar Porrini Prandini, Aurora Mustaccia, Valentina Fogazzi, Marta Allesina, Marco Krengli, Renzo Boldorini

**Affiliations:** 1 Dipartimento di Scienze della Salute, Scuola di Medicina, Università del Piemonte Orientale, Via Solaroli 17, 28100 Novara, Italy; omarprandiniporrini@gmail.com (O.P.P.); mustaccia.aurora@hsr.it (A.M.); valentina.fogazzi@gmail.com (V.F.); marta.allesina@uniupo.it (M.A.); renzo.boldorini@uniupo.it (R.B.); 2 Centro Interdipartimentale di Ricerca Traslazionale Sulle Malattie Autoimmuni & Allergiche (CAAD), Università del Piemonte Orientale (UPO), Corso Trieste 15A, 28100 Novara, Italy; 3 Unità di Radioterapia, Dipartimento di Medicina Traslazionale, Università del Piemonte Orientale, Via Solaroli 17, 28100 Novara, Italy; marco.krengli@uniupo.it; 4 Unità di Radioterapia Oncologica, Azienda Ospedaliera Universitaria Maggiore della Carità, Corso Mazzini 18, 28100 Novara, Italy; 5 Unità di Patologia, Azienda Ospedaliera Universitaria Maggiore della Carità, Corso Mazzini 18, 28100 Novara, Italy

**Keywords:** meningiomas, telomerase reverse transcriptase, molecular markers, ALT, prognosis

## Abstract

Background: The role of telomerase reverse transcriptase (TERT) gene promoter mutations (*pTERT*) in atypical and anaplastic meningiomas remains controversial. This study aimed to evaluate their impact on the histologic diagnosis and prognosis in a retrospective series of 74 patients with atypical and anaplastic meningioma, including disease progression and relapse. A supplementary panel of 21 benign tumours was used as a control cohort. Materials and Methods: The mutation rate of the *pTERT* gene was assessed by Sanger sequencing. ATRX protein expression was detected by immunohistochemistry. The phenotypic and genotypic intra-tumour heterogeneity was studied in a sub-group of 12 cases using a Molecular Machines & Industries (MMI) CellCut laser microdissection (LMD) system. Results: *pTERT* mutations were detected in 12/74 (17.6%) malignant meningiomas. The mutation rate was significantly higher in anaplastic meningiomas (7/23, 30.4%) compared to atypical tumours (5/48, 10.4%) (*p* = 0.0443). In contrast, the mutation rate was < 5% in benign tumours. All *pTERT* mutant cases retained nuclear ATRX immunoreactivity. *pTERT* mutations were significantly associated with the histologic grade (*p* = 0.0443) and were adverse prognostic factors for anaplastic tumours (*p* = 0.06). Conclusion: We reported on the *pTERT* mutation spectrum in malignant meningiomas, supporting their use in the prognostic classification.

## 1. Introduction

Meningiomas are the most common primary tumours of the central nervous system (CNS) [1]. The majority of meningiomas are low-grade (WHO grade I) tumours that can be effectively managed by stereotactic radiosurgery. A subset of tumours (WHO grade II–III) displays malignant histologic features, risk of recurrence and poor Progression-Free Survival (PFS) despite optimal management, including surgery +/− radiotherapy [2,3,4,5].

According to the current CNS tumour classification system [1], meningiomas are classified into three main groups that differ in degree and biological behaviour. Grade I meningiomas are benign tumours with <4 mitoses per 10 high-power fields (HPF) and include various histologic subtypes without clinical significance, see pp. 230–245 in [1].

Grade II (atypical) meningiomas are classified as an intermediate grade between benign and malignant forms. They comprise atypical, clear cells and chordoid morphologic variants and show increased mitotic activity (with ≥4 mitoses per 10 HPF), brain infiltration or ≥3 of the histopathologic features associated with atypia (increased cellularity, small cell areas with a high nucleus-cytoplasm ratio, prominent nucleoli, sheet-like growth, and foci of spontaneous necrosis) [1].

Grade III (anaplastic) meningiomas show malignant cytology with high mitotic activity (i.e., ≥20 mitoses for 10 HPF), extensive foci of necrosis, Ki-67 cell proliferation index > 20%, and anaplasia [1,6]. They incorporate papillary, rhabdoid and anaplastic subtypes, which inevitably recur within ten years. The prognosis is poor, with median survival times ranging from <2 and >5 years, based on the extent of resection [1].

The significant impact of the 2016 WHO revised classification system affects grade II meningiomas, as brain infiltration became one of the diagnostic criteria [7]. Identification of new objectives and non-operator dependant criteria is essential for the clinical decision-making process and follow-up (FU) of patients with meningioma, and additional molecular studies are necessary to predict the risk of recurrence and radiosensitivity [8,9,10].

In most cases, meningiomas can be removed entirely, but atypical and anaplastic tumours frequently recur [1]. From a prognostic point of view, the clinical outcome of the atypical and anaplastic meningioma is influenced, respectively, by the recurrence rate and the overall survival (OS) time [1] and pp. 230–245 in [1]. The major clinical factor in recurrence is the extent of resection, which is influenced by tumour site, extent of invasion, attachment to vital intracranial structures, and by the surgeon’s skill.

From a molecular perspective, the genetic model for tumourigenesis and progression in meningioma involves the stepwise accumulation of genetic and cytogenetic aberrations, besides gene expression changes [1,11,12]. The progression from benign (grade I) to atypical (grade II) and then anaplastic (grade III) tumours is triggered by the acquisition of genetic mutations in the promoter region of the *telomerase reverse transcriptase* (*pTERT*) gene [10,13,14]. The enzyme “telomerase” catalyzes the addition of repetitive non-coding DNA (TTAGGG) nucleotide sequences to the end of eukaryotic chromosomes and prevents degradation of chromosomal ends.

With subsequent maintenance of the telomere length, the reactivation of telomerase is essential in tumourigenesis because it contributes to tumour cell immortalization in high-grade solid tumours, including meningioma [15,16].

Two hotspot point mutations in the human *pTERT* gene (chr5; 1,295,228 (C228T) and chr5; 1,295,250 (C250T)) contribute to the telomerase reactivation. Both give origin to de novo binding sites for transcription factors that belong to the ETS/TCF family and are associated with an increased gene expression from two to four times [17,18]. After they were first described in melanoma, their presence has been identified in several solid tumours, including CNS tumours, and correlated to malignant transformation and unfavourable patient survival [19].

In meningiomas, previous studies reported *pTERT* mutations in malignant tumours related to tumour progression, increased risk of recurrence, and poor prognosis [10,13,20]. In atypical and anaplastic tumours, they are negative prognostic factors significantly associated with shorter Time-to-Progression (TTP) and OS [10,20]. The presence of *pTERT* mutations directly correlates to a significant upregulation of *TERT* gene expression and telomerase activity [16,21]. In contrast, *pTERT* mutations are rare genetic events in benign meningiomas.

In the absence of telomerase, tumours may exploit a telomerase-independent mechanism for the telomere elongation known as ALT (Alternative Length Telomerase) phenotype, which is associated with inactivating mutations in *ATRX* or *DAXX* genes. The ATRX, protein also known as ATP-dependent helicase ATRX, is involved in transcriptional regulation and chromatin remodelling and is encoded by the *ATRX* gene. *ATRX* mutations are deleterious genetic events because they give origin to truncated or inactive ATRX proteins. The ALT phenotype has been widely reported in many cancers (i.e., gliomas, astrocytomas, osteosarcomas, and neuroendocrine tumours of the pancreas) [22,23], but poorly in meningiomas.

To date, the knowledge of the molecular mechanism driving the malignant progression in meningioma remains poor, and the molecular landscape in meningioma needs to be further investigated.

The aims of this study were: (i) to evaluate the impact of the revised WHO classification system on a retrospective series of 83 samples from 74 patients, including 37 grade II (atypical) and 42 grade III (anaplastic) tumours and 4 tumours in differential diagnosis between grade II and III, and 21 benign tumours; (ii) to investigate the mutation spectrum of the human *pTERT* gene and the ATRX protein expression; (iii) to study the phenotypic and genotypic intra-tumour heterogeneity in a sub-group of selected tumours using a Molecular Machines & Industries (MMI) CellCut laser microdissection (LMD) system.

## 2. Materials and Methods

### 2.1. Brain Tumour Specimens

#### 2.1.1. Sample Collection

A total of 83 cases with a histologic diagnosis of atypical and anaplastic meningiomas were retrospectively collected between 1999 and 2019 from the archive of the Unit of Pathology—Maggiore della Carità Hospital/University of Eastern Piedmont (Novara, Italy). The initial diagnosis was in agreement with the World Health Organization (WHO) guidelines [1,24,25].

Patients underwent partial or total resection at the Neurosurgery Unit. The surgical resection radicality was evaluated using the Simpson grade, by grouping the classifications from one to three as gross total resection (GTR) and from four to five as subtotal resection (STR) [26].

Surgical specimens were formalin-fixed, paraffin-embedded (FFPE), and cut in 3 μm-thick serial sections. Clinical and FU data were recorded.

An independent control cohort of 21 cases, diagnosed as grade I meningioma, was collected from the Neuro-bio-oncology Center/Fondazione Policlinico di Monza (Vercelli, Italy).

#### 2.1.2. Patient Stratification

Radiation therapy (RT) was administered at different radiotherapy units through Piedmont and Lombardy, but most of the patients were referred to the radiotherapy unit of Novara (Italy).

RT was recorded for 32 patients (one with WHO 2016 grade I meningioma, 14 with atypical, and 17 with anaplastic meningioma). Among them, 14 (43.8%) were alive at the last FU. Five patients underwent stereotactic radiosurgery (SRS) by Gamma Knife^®^ or CyberKnife^®^ and received a variable dose of RT (range 14–16 Gy); the remaining 27 underwent GTR and were scheduled with fractionated volumetric-modulated arc therapy (VMAT) or intensity-modulated radiation therapy (IMRT), with an average total dose of 56.3 Gy (range 50.4–66).

The tumour location was scored according to the WHO International Classification of Disease for Oncology 3 (ICD-O-3) [27].

#### 2.1.3. Ethics Statement

Human brain specimens were obtained and used in compliance with the local institutional review board and Committee on Human Research and with the ethical human subject principles of the World Medical Association Declaration of Helsinki Research.

### 2.2. Immunohistochemistry (IHC)

Besides haematoxylin and eosin (H & E) staining, IHC on the FFPE blocks were performed using a Ventana Full BenchMark^®^ XT automated immunostainer (Ventana Medical Systems, Inc., Tucson, AZ, USA). The UltraViewTM Universal DAB Detection Kit (Ventana) was the detection system. Heat-induced epitope retrieval (HIER) was obtained with Cell Conditioning solution (CC1Tris-based EDTA buffer, pH 8.0, Ventana). Negative controls were obtained by omission of the primary antibody. Primary antibodies used were: CONFIRM™ anti-Ki-67 (clone 30-9) rabbit monoclonal antibody (pre-diluted, Ventana), anti-ATRX rabbit polyclonal antibody (HPA001906, 1:400 diluted, Sigma-Aldrich, Saint Louis, MO, USA), and anti-glial fibrillary acidic protein (GFAP) mouse monoclonal antibody (M0761, 1:200 diluted, Dako, Jena, Germany).

The Ki-67 labelling index (LI) was calculated as the average percentage of positive nuclei in hotspot areas at the maximum LI on a total number of 1000 cells at 1000× magnification using Image-Pro Plus 2.0 Analysis Software (Media Cybernetics, Inc. Rockville, MD, USA). Images were examined and acquired on a Leica Wild Leitz Diaplan fluorescence phase-contrast trinocular microscope equipped with a Leitz 4 × 5″ microscope camera (Leica Microsystems GmbH, Wetzlar, Germany).

IHC for ATRX was used as a surrogate marker for the mutation status of the *ATRX* gene [28]. A threshold of 5% of positive tumour nuclei was used to assign immunopositivity for ATRX [29].

IHC for GFAP was used to detect reactive astrocytosis, featured by entrapped islands of GFAP-positive parenchyma at the periphery of the tumour in the presence of invasion into brain parenchyma.

The mitotic index (MI) was calculated as the average number of mitoses in ten random HPF.

### 2.3. Laser Microdissection (LMD)

Laser microdissection was performed with a Molecular Machines & Industries (MMI) CellCut LMD system (MMI GmbH, Eching, Germany) coupled with an Eclipse TE-2000 fluorescent microscope (Nikon Instruments, Melville, NY, USA). Selected areas were cut from the tissues by a UV laser beam. In order to maintain the integrity of cell clusters and preserve DNA from the heat of the laser, we took care to leave a space between the laser circle and the cell limit (approximately 5 µm). Microdissected cells were then collected on an adhesive lid of a microtube placed on the area. The gaps in the tissue visually confirmed the success of the cell capture after lid removal.

### 2.4. Molecular Genetics

#### 2.4.1. DNA Extraction

Genomic DNA (gDNA) from FFPE tumour samples was isolated using the QIAamp DNA FFPE Tissue Kit (Qiagen NV, Venlo, The Netherlands). The tumour cell content was verified on H & E- stained sections. Only specimens containing >90% neoplastic cells were used for the DNA isolation. gDNA was qualified and quantified using a NanoDrop^TM^ 2000 spectrophotometer (Thermo Fisher Scientifics Inc., Waltham, MA, USA).

#### 2.4.2. pTERT Mutation Analysis

One primer pair designed on gDNA was used to amplify by polymerase chain reaction (PCR) the core promoter region of the human *TERT* gene (GenBank accession no. NM_198253) from −203 to −46 bp upstream of the start codon. The PCR product contains the two hotspot mutations C250T (−146 bp) and C228T (−124 bp).

The primer sequences are available on demand. PCR amplification was performed in a total volume of 12.5 μL containing 50 mM KCl, 10 mM Tris–HCl (pH 8.3), 1.5 mM MgCl_2_, 250 μM of each dNTP, 0.365 U of AmpliTaq Gold^®^ 360 DNA polymerase (Thermo Fisher Scientifics Inc.), 10 pmol of each primer and 50 ng of gDNA. The following touchdown PCR protocol was used: initial denaturation at 96 °C for 10 min, followed by 94 °C for 30 s, 65 °C to 55 °C for 30 s with a decrement of 0.5 °C per cycle for 20 cycles, and 72 °C for 30 s. The additional 25 cycles were at 94 °C for 30 s, 55 °C for 30 s, and 72 °C for 30 s. A final elongation step of 10 min at 72 °C was added.

#### 2.4.3. Sanger Sequencing

Amplicons were analyzed by Sanger direct sequencing using the BigDye^®^ Terminator v1.1 Cycle Sequencing Kit (Thermo Fisher Scientifics Inc.). Data were collected by the Sequencing Analysis v.5.3.1 software (Thermo Fisher Scientifics Inc.). All the identified sequence variations were confirmed with at least two independent PCR and sequencing experiments. Mutation nomenclature is in agreement with the current Human Genome Variation Society guidelines (http://varnomen.hgvs.org/; accessed on 16 June 2019). The reported nucleotide and amino acid numbering are relative to the transcription start site (+1), corresponding to the A of the ATG on the relevant GenBank reference sequence. The somatic origin of each putative sequence variation was verified by the analysis of the patient constitutional DNA.

### 2.5. Statistical Methods

Associations between categorical variables were evaluated using 2 × 2 contingency tables and the Chi-square (χ^2^) or the two-tailed Fisher’s exact test, as appropriate. The Student’s *t*-test was used to compare quantitative variables (age at onset).

Disease progression (DP) was defined as clinic progression of a tumour from a lower to a higher histologic grade. Recurrence (R) was defined as the reappearance of the tumour, later in time, with the same histologic grade as the previous one.

OS was defined as the time (months) from the histologic diagnosis (after reclassification according to 2016 WHO criteria) or surgery, and the patient’s death or last FU. Patients alive at the last FU were considered censored events.

The TTP was estimated in the sub-group of atypical and anaplastic tumour of which all disease progressions or recurrences were genotyped. It was calculated as the time (months) from the first surgery to the first documented disease progression or recurrence date.

Survival curves were estimated using the Kaplan–Meier method and compared by the log-rank test (Mantel-Cox).

Statistical analysis was carried out by SPSS v25.0 software (IBM Corp., Armonk, NY, USA). *p*-values < 0.05 were considered statistically significant.

## 3. Results

### 3.1. Patient Demographics

Seventy-four patients (38 males and 36 female) have been included in the study (male-to-female ratio 1.05:1). Nine of these underwent more than one surgery, because of tumour regrowth: one patient had three relapses, one had two relapses and six one relapse.

All patients were Caucasian, except for one African lady. The median age at diagnosis was 67 (range 23–85).

### 3.2. Histopathologic Reclassification According to 2016 WHO Guidelines

All cases underwent a histologic reclassification according to the current WHO guidelines [1] by two expert neuropathologists (R.B. and O.P.P.). The revision of the histologic diagnosis was based on the cell proliferation index Ki-67, MI, and on the presence of tumour infiltration into the brain parenchyma [1].

At the initial diagnosis, the series was originally composed of 83 samples from 74 patients: 37 grade II (atypical) and 42 grade III (anaplastic) meningiomas, 2 cases initially classified as differential diagnosis between grade II and III tumours, and 1 case originally classified between grade I and II tumour. One specimen was unsuitable for the genetic analysis due to the presence of post-surgical artefacts.

After the diagnostic reclassification, the initial diagnosis changed in 17 of 79 (21.5%) cases. Out of 42 tumours initially classified as anaplastic, 15 (35.7%) were reclassified as atypical meningioma due to the absence of all the histologic features required according to the current WHO guidelines. In contrast, the initial diagnosis of atypical meningioma was confirmed in 35 of 37 (94.6%) cases. The three cases in differential diagnosis were all reclassified as benign tumours.

In agreement with the new classification, the series was composed of 27 grade III tumours, 50 grade II tumours, and three reclassified as grade I meningiomas that were added to the supplementary panel of 21 grade I tumours.

In addition, we included 21 cases of disease progression or relapse from 19 patients with WHO grade II or III. In three cases, the tumour relapsed without histologic progression (14.3%). Conversely, 18 cases showed progression to a higher histologic grade: six cases from grade I to II (33.3%), eight cases from grade II to III (44.4%), and four cases from grade I to III (22.2%) at different time points.

### 3.3. Molecular Genetics

#### 3.3.1. pTERT Mutation Analysis in Brain Tumour Specimens

The *pTERT* mutation status was assessed in 82 samples from 74 patients with malignant tumours (including disease progression or relapses) and in 21 samples from benign tumours. Eight malignant cases were evaluated in two different histologic areas due to morphologic heterogeneity.

The mutation status of the *pTERT* gene was successfully determined in 95/101 cases (94.1%) (Table 1). Overall, in malignant meningiomas from the initial series *pTERT* activating mutations were detected in 13/74 (17.6%) cases (Table 1).

Of the 13 mutant samples, seven (53.8%) were diagnosed as grade III tumours and five (38.5%) as grade II. The mutation rate was significantly higher in grade III tumours (7/23, 30.4%) compared to grade II tumours (5/48, 10.4%) (*p* = 0.0443, Fisher’s exact test).

In the control cohort of grade I tumours (*n* = 24), a C228T *pTERT* mutation (4.2%) was detected in only one patient that underwent a disease relapse.

The C228T point mutation was identified in 8/13 (61.5%) cases, whereas the C250T substitution in 5/13 (38.5%) cases (Table 1). All mutations were mutually exclusive and of somatic origin.

All *pTERT* mutant cases retained nuclear ATRX immunoreactivity.

Immunophenotypic and molecular features of *pTERT* mutant cases are described in Table 2.

#### 3.3.2. Relationship of pTERT Mutations with Clinical and Molecular Features

In order to study the genotype–phenotype correlations referred to the *pTERT* mutation status, we compared the following clinic–pathologic variables: histologic grade and subtype, tumour location, gender, age at onset, disease progression, and recurrence.

*pTERT* mutations were significantly associated with the histologic malignancy grade, which were detected in grade III tumours (7/23, 30.4%) significantly more frequently than in grade II tumours (5/48, 10.4%) (*p* = 0.0443, Fisher’s exact test).

Among the 13 mutant cases, malignant histology was common: six cases were anaplastic (WHO grade III), five atypical (WHO grade II), and one case showed a “clear cell” appearance (WHO grade II). Only one case had transitional histology (WHO grade I) (Table 2).

The average age at onset was 63.5 years (range 23–80) in *pTERT*-mutant cases (including cases that developed a *pTERT* mutation during disease progression and/or relapse) and 69 years (range 32–84) in wild type patients (*p* > 0.05, Student’s *t*-test).

All cases with a histologically documented disease progression from grade I/II to grade III harboured a *pTERT* mutation, but not from grade I to grade II.

Although the acquisition of *pTERT* mutations drives tumour progression towards grade III, the mutation rate in de novo anaplastic tumours (3/9, 33.3%) was similar to that observed in anaplastic tumours secondary to a malignant progression (4/12, 33.3%).

In the mutant group, six cases were at new-onset, four disease progression (two from grade II to III and two from grade I to III), and three recurrences (two diagnosed as grade II and one as grade I).

Two of the mutant cases (both grade III tumours) had lung metastases (Table 2). The former harboured a C228T *pTERT* mutation and developed a disease progression in a few months; the latter arose as a de novo malignant tumour with a C250T *pTERT* mutation.

### 3.4. IHC

In malignant tumours, all but two cases (80/82, 97.6%) showed a diffuse nuclear immunoreactivity for ATRX in tumour cells. ATRX-positive nuclei of endothelial cells (ECs) and infiltrating lymphocytes were positive internal controls for the immunohistochemical reaction.

The two cases with loss of immunoreactivity for ATRX were *pTERT* wild type.

### 3.5. Phenotypic and Genotypic Intra-Tumour Heterogeneity

The phenotypic and genotypic intra-tumour heterogeneity was studied in 25 selected areas from a sub-group of 12 cases, including seven atypical, four anaplastic, and one “clear cell” tumour according to the revised classification (Supplementary Table S1). Nineteen withdrawals were obtained through manual macro-dissection and six using the MMI CellCut LMD system (Supplementary Table S2). By this latter, we collected and pooled approximately fifteen tubes for sample (from 10 to 20 tubes) to proceed to the DNA isolation. A similar integrated surface (range 2,555,109.7–5,104,412.0, average 3,922,783.7 µm^2^) was collected to standardize all samples (Supplementary Table S2).

For each case, at least two different tumour areas were selected according to the presence of morphologic intra-tumour heterogeneity (i.e., histologic grade, Ki-67 cell proliferation index, tumour infiltration into the brain parenchyma) (Supplementary Table S2).

**Figure 1:**
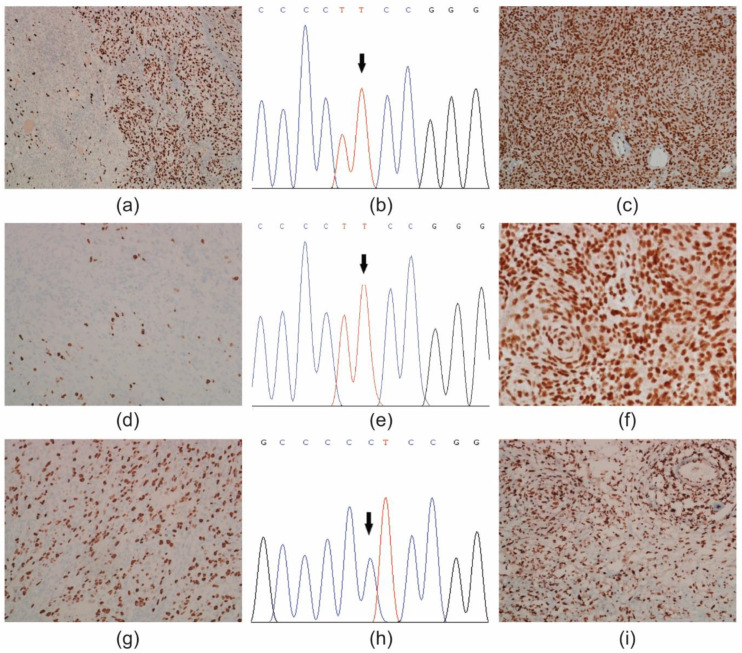
Intra-tumour heterogeneity in a WHO grade II (atypical) meningioma (Case #7). (**a**) Primary tumour, Ki-67 cell proliferation index; DAB, original magnification (OM) × 200. (**b**) Electropherogram of a C250T *pTERT* mutation (chr5; 1,295,250). Arrow indicates the position of the nucleotide substitution. (**c**) ATRX immunoreactivity was retained in the nuclei of tumour cells; DAB, OM × 200. (**d**) Withdrawal A, low Ki-67 cell proliferation index; DAB, OM × 200. (**e**) Electropherogram of a C250T mutation (chr5; 1,295,250). (**f**) ATRX immunoreactivity in the nuclei of tumour cells; DAB, OM × 400. (**g**) Withdrawal B, high Ki-67 cell proliferation index; DAB, OM × 200. (**h**) Electropherogram of a wild type *pTERT* gene promoter (chr5; 1,295,250). (**i**), ATRX immunoreactivity in the nuclei of tumour cells; DAB, OM × 200. DAB, 3,3′-Diaminobenzidine.

**Figure 2:**
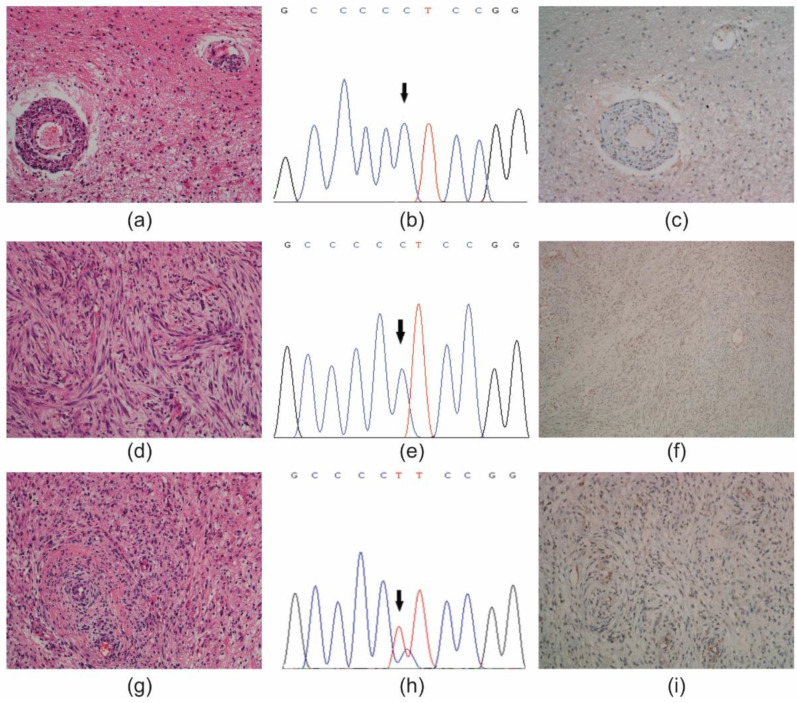
Intra-tumour heterogeneity in a WHO grade III (anaplastic) meningioma (Case #15). (**a**) Primary tumour, mild infiltration; hematoxylin & eosin (H & E) staining; original magnification (OM) × 200. (**b**) Electropherogram of a wild type *pTERT* gene promoter (chr5; 1,295,228). Arrow indicates the position of the mutation hotspot. (**c**) Loss of ATRX immunoreactivity in the nuclei of tumour cells; DAB, OM × 200. (**d**) Withdrawal A, absence of tumour infiltration; H & E, OM × 200. (**e**) Electropherogram of a wild type *pTERT* gene promoter (chr5; 1,295,228). (**f**) Loss of ATRX immunoreactivity in the nuclei of tumour cells; DAB, OM × 200. (**g**) Withdrawal B, tumour infiltration in the brain parenchyma; H & E, OM × 200. (**h**) Electropherogram of a C228T *pTERT* mutation (chr5; 1,295,228). (**i**) Loss of ATRX immunoreactivity in the nuclei of tumour cells; DAB, OM × 200. DAB, 3,3′-Diaminobenzidine.

By comparing phenotypic and genotypic features of the different tumour areas (i.e., *pTERT* mutation status and ATRX immunoreactivity) with the matched primary solid tumour, two cases showed evidence of intra-tumour heterogeneity (Figure 1 and Figure 2) (Supplementary Tables S2 and S3).

The first case diagnosed as anaplastic meningioma (Case #7), was selected based on the presence of heterogeneity for the Ki-67 cell proliferation index in two different areas (Figure 1a–i). The primary tumour harboured a C250T *pTERT* mutation and retained ATRX nuclear expression in tumour cells (Figure 1a–c). The withdrawal A (from a manual microdissection), showed a Ki-67 LI = 19.3%, tumour cells with a C250T *pTERT* mutation and nuclear ATRX immunoreactivity (Figure 1d–f). The withdrawal B (from a manual macrodissection) had a Ki-67 LI = 36% and *pTERT* wild type tumour cells with retained ATRX expression (Figure 1g–i).

The second case diagnosed as atypical meningioma (Case #15), was selected based on the presence or absence of tumour infiltration into the brain parenchyma (Figure 2a–i). The primary tumour showed a mild tumour infiltration and loss of ATRX expression in *pTERT* wild type tumour cells (Figure 2a–c). The withdrawal A (from LMD), besides the absence of tumour invasion, displayed loss of ATRX expression in *pTERT* wild type tumour cells (Figure 2d–f). The withdrawal B (from a manual macrodissection) had a Ki-67 LI = 13% and showed loss of ATRX expression in tumour cells that harboured a C228T *pTERT* mutation (Figure 2g–i). Initially classified as grade III meningioma, the diagnosis was then replaced as grade II (atypical) meningioma based on the presence of tumour infiltration into the parenchyma, MI = 8 and Ki-67 < 20 [1].

### 3.6. Survival Analysis

The relationship between *pTERT* mutations and OS was analyzed in a series of 60 meningiomas with recorded FU. The remaining cases of the series were excluded from the survival analysis due to reclassification as grade I tumours (*n* = 3), intraoperative death (*n* = 3), or loss at FU (*n* = 7). The control cohort of grade I meningioma was not considered.

As of June 2019, of the 37 patients diagnosed with grade II meningioma, 19 died (51.3%) and 18 lived (48.6%). Among grade III tumours (*n* = 23), 15 were deceased (65.2%) and eight were alive (34.8%). In total, 26 of the 60 patients were still alive (43.3%). The median OS among all cases was 49 months (I.C. 95% = 8.03–89.8).

**Figure 3:**
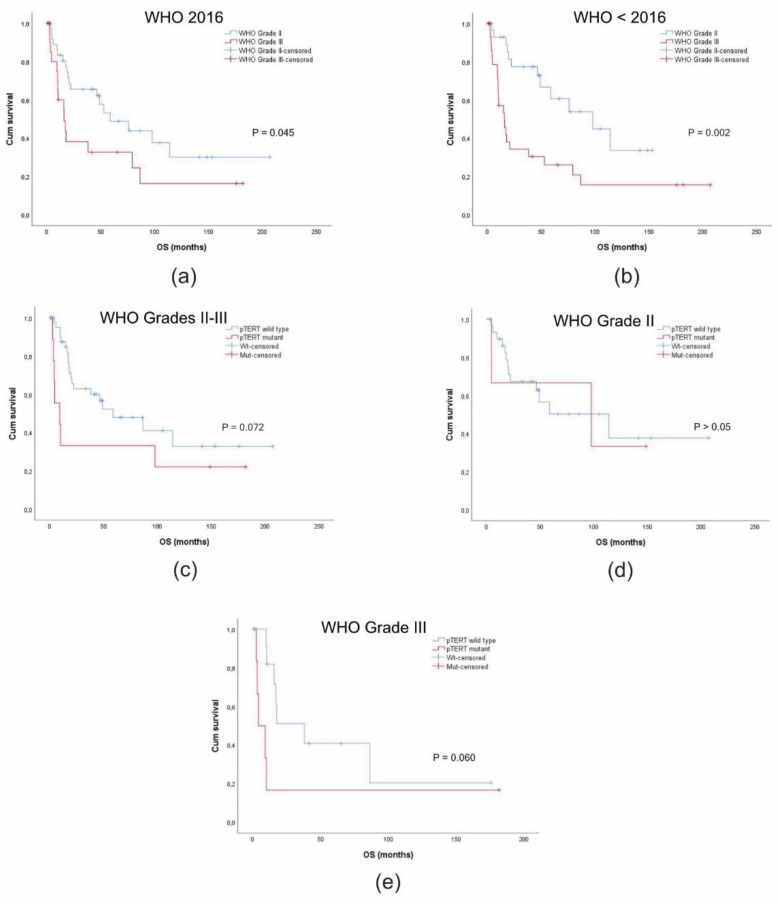
Survival analysis in atypical and anaplastic meningiomas. (**a**) Kaplan–Meier survival curves for overall survival (OS) in 60 cases with malignant meningioma diagnosed according to the current WHO CNS classification system [1]. Censored cases were 18/37 (48.6%) in WHO grade II tumours and 8/23 (34.8%) in WHO grade III tumours. (**b**) Kaplan–Meier survival curves for OS in 60 cases with malignant meningioma classified according to previous WHO CNS classification systems (<2016). Censored cases were 17/29 (58.6%) in WHO grade II tumours and 9/31 (29.0%) in WHO grade III tumours. (**c**) Kaplan–Meier survival curves for OS in 53 cases according to the *pTERT* mutation status. Censored cases were 24/44 (54.5%) in *pTERT* wild type cases and 2/9 (22.2%) for *pTERT* mutant cases. (**d**) Kaplan–Meier survival curves for OS in 20 cases according to the *pTERT* mutation status in WHO grade II tumours. Censored cases were 17/30 (56.7%) in *pTERT* wild type cases and 1/3 (33.3%) in *pTERT* mutant cases. (**e**) Kaplan–Meier survival curves for OS in 20 cases according to the *pTERT* mutation status in WHO grade III tumours. Censored cases were 7/14 (50%) in *pTERT* wild type cases and 1/6 (16.7%) in *pTERT* mutant cases.

The median OS was significantly lower in the sub-group of grade III meningiomas (15.7 months; C.I. 95% = 6.5–24.9) compared to the sub-group of grade II tumours (58.6 months; C.I. 95% = 22.7–94.6) (χ^2^ = 4.011, *p* = 0.045, log-rank test) (Figure 3a). The difference in the median survival time was highly more significant according to the previous WHO CNS classification systems. The median OS was 97.6 months (C.I. 95% = 36.4–158.7) in the tumour sub-group of grade II tumours and 15.5 months (C.I. 95% = 8.1–89.8) in that of grade III tumours (χ^2^ = 9.551, *p* = 0.002, log-rank test) (Figure 3b).

In the genotyped tumour sub-group, the total number of patients considered for the survival analysis was 53 cases (nine *pTERT* mutant and 44 wild type). In the former, the mortality rate at the last FU was 7/9 (77.8%) with a median survival time of 9.2 months (C.I. 95% = 0.1–22.7) (Figure 3c). In the latter, it accounted for 20/44 (45.5%) with a median OS of 58.6 months (C.I. 95% = 13.4–103.8). The difference in the median survival time showed a borderline significance *(*χ^2^ = 3.248, *p* = 0.072, log-rank test) (Figure 3c). Notably, two *pTERT* mutant cases were long-term survivors, i.e., young patients (age at onset < 45 yrs) who underwent GSR and complete schedule of RT (Table 1). Removing these two cases, the difference between the two sub-groups was highly significant *(*χ^2^ = 17.728, *p* = 0.0001, log-rank test) (data not shown).

Because of the low number of *pTERT* mutant cases (*n* = 3) with recorded FU in the sub-group of atypical tumours (*n* = 20), the survival analysis was not informative (Figure 3d). In contrast, *pTERT* mutations were negative prognostic factors for OS in the tumour sub-group of anaplastic meningiomas (χ^2^ = 3.550, *p* = 0.060, log-rank test) (Figure 3e). We could not evaluate their possible prognostic impact in terms of TTP due to the low number of cases with disease progression.

## 4. Discussion

In the present study, we histologically reclassified a retrospective series of 83 atypical and anaplastic meningiomas according to the current WHO guidelines [1] to evaluate the impact of the revised classification system.

The revision of the histologic diagnosis resulted in a significant change in the histopathologic classification in the sub-group of tumours initially classified as anaplastic (grade III) meningioma. Thirty-seven per cent of these tumours were reclassified as atypical (grade II) based on the absence of all the histologic features required by the new criteria. Conversely, the initial diagnosis of atypical meningioma was confirmed in 94.6% of the cases.

The current criteria of the WHO classification for meningiomas are based on the evaluation of morphologic aspects that are objective (i.e., MI and infiltration into brain parenchyma) and subjective (i.e., the definition of atypia and presence or absence of necrosis, which can be a consequence of surgical or preoperative procedures). All these features are prone to inter- and intra-observer variability, which can affect the histologic diagnosis.

From a prognostic point of view, the more stringent criteria for the diagnosis of malignant meningioma in the current WHO classification system impacts significantly on the survival outcomes compared to the previous one.

The series analyzed in this study is numerically consistent, considering that the incidence for atypical and anaplastic meningioma in literature is 18–30% for the former and 1–3% for the latter [11].

Previous studies have evaluated the molecular features of these neoplasms, focusing mainly on the molecular pathways related to the maintenance of telomere length, as well as to various genes involved in cell survival or apoptosis or in the control of invasiveness (*SMO*, *TRAF7*, *AKT1*, *KLF4,* and *SMARCB1*) [30,31,32]. As in brain tumours of astroglial derivation/differentiation, in which gene mutations of *TERT* or *ATRX* genes are significantly involved in carcinogenesis, we analyzed the role of *pTERT* mutations and ATRX protein expression in malignant meningiomas to assess their actual mutation rate and their possible prognostic impact in terms of OS.

Activation of telomerase by *pTERT* mutations is frequent in meningiomas and associated with the histologic grade [33]. Whereas rare in benign tumours, their frequency increases significantly from grade II to grade III tumours.

In the present series, we found an overall frequency of *pTERT* mutations in malignant meningiomas equal to 17.6%, in line with the literature [10]. The mutation rate is significantly higher in anaplastic meningiomas (30.4%) compared to atypical meningiomas (10.4%) and statistically associated with the histologic grade (*p* = 0.0443). This finding confirms that this genetic alteration is a critical carcinogenic event associated with higher degrees of anaplasia and a more aggressive biological behaviour.

*pTERT* mutations prevail in tumours with a clear-cut malignant histology or a “clear cell” appearance (WHO grade II). According to the new WHO guidelines, one case only showed a benign (grade I) transitional histology; this tumour, initially diagnosed as atypical meningioma, was further reclassified as a benign tumour. This could be an emblematic case: a tumour with Ki-67 LI = 14%, a maximum MI = 2 among the sections evaluated, and the presence of necrosis and intra-tumour heterogeneity with small cell areas, with documented relapse in 2016.

Remarkably, *pTERT* mutations affect younger patients (average age at onset of 63.5 years, range 23–80), but the difference between *pTERT* mutant and wild type cases is not statistically significant.

Finally, *pTERT* mutations are present in all patients who underwent a malignant histologic progression (from grade I or II to grade III). However, the observed mutation rate in de novo anaplastic tumours (3/9, 33.3%) is similar to that observed in anaplastic tumours secondary to a malignant progression (4/12, 33.3%). This finding confirms previous observations about the *pTERT* mutation rate in primary and secondary grade III meningiomas [34]. Conversely, no case in progression from grade I to II harboured a *pTERT* mutation. Tumours showing relapse without histologic progression did not harbour *pTERT* mutations.

In malignant meningiomas, all tumour cells retained nuclear ATRX expression; likewise, non-tumour cells such as ECs, microglia/macrophages, reactive astrocytes, and lymphocytes. Immunoreactivity for ATRX was detected in all cases, except for two. Loss of immunopositivity is inversely correlated to the presence of *pTERT* mutations, as described in the literature for other tumour types [23,29]. The low prevalence of the loss of ATRX suggests that this alternative mechanism of telomere elongation is a rare phenomenon in malignant meningiomas [23]. The active telomere maintenance in malignant meningiomas is due to the presence of recurrent *pTERT* mutations.

By manual macrodissection or LMD, we studied the phenotypic and genotypic intra-tumour heterogeneity in 25 spatially distinct tumour areas from 13 malignant meningiomas. Our data reveal regional alterations and tumour heterogeneity in two cases (one atypical and the other anaplastic), in agreement with previous studies in the literature [35,36], that the biological behaviour could explain. Recently, a spatial heterogeneity at molecular and radiographic level has been referred by single-cell analysis to differential clonal transcriptomic, epigenomic, and histopathologic signatures [36].

Interestingly, *pTERT* mutations are adverse prognostic factors in terms of OS in the overall group of malignant meningiomas and, more significantly, in the sub-group of anaplastic tumours.

Since they appear to be correlated to a disease progression in terms of higher tumour grade, we suggest that mutation analysis of *pTERT* needs to be added, as ancillary test, to the histologic diagnosis in all cases with atypical and anaplastic features, even those without the WHO criteria [37].

Finally, the discovery in our series of two cases of anaplastic meningioma, both of them harbouring *pTERT* mutation, with metastatic lung disease, cytologically and radiologically demonstrated, suggests a possible predictive role for these rare events [38].

The present study describes a comprehensive analysis of *pTERT* mutations in a large retrospective series of 83 atypical and anaplastic meningiomas histologically revised according to the current WHO guidelines [1]. We provide results about the mutation rate and their associations with OS; however, we could not determine if *pTERT* mutations were independent prognostic factors in malignant meningiomas due to the low number of cases with recorded FU.

## 5. Conclusions

*pTERT* mutations are pivotal genetic events during the malignant progression of meningiomas. They could be used as biomarkers to identify meningiomas at risk of malignant transformation and worse clinical outcomes.

## Figures and Tables

**Table 1:** *pTERT* mutation rate according to the 2016 WHO classification system.

Histology	Patients * (*n*)	Mutation Rate C250T C228T *n* (%)			Average Age at Onset
Meningioma	24	1/24 (4.2)	1 (100)	0 (0)	64.0
Atypical meningioma	50	5/48 (10.4)	1 (20)	4 (80)	65.3
Anaplastic meningioma	27	7/23 (30.4)	3 (42.9)	4 (57.1)	65.6

Abbreviations: WHO, World Health Organization; C250T, (chr5; 1,295,250 (−146 upstream of the transcription start codon)); C228T, (chr5; 1,295,228 (−124 upstream of the transcription start codon)). * Histologic classification according to the revised WHO classification CNS system [1].

**Table 2:** Immunophenotypic and molecular features of the cases with *pTERT* mutation are classified according to the WHO 2016 guidelines.

Case #	*pTERT* Genotype	ATRX	Gender	Age at Onset	Anatomic Location	Morphologic Variant	Mitotic Index	Ki-67 LI (%)	WHO Grade 2016	Surgery	DP/R	Metastases	OS
1	C250T	+	M	80	Frontal	Transitional	2	14	I	2	R	-	A
2	C228T	+	F	77	Sickle	Atypical	8	23	II	3	R	-	4.5
3	C228T	+	F	70	Sickle	Atypical	5	29	II	1	-	-	4.2
4	C250T	+	M	45	Frontal	Atypical	10	30	II	2	R	-	118
5	C228T	+	M	66	Frontal	Atypical	12	20	II	1	-	-	NA
6	C228T	+	F	23	Cauda equina	Clear cell	3	10	II	1	-	-	148.5
7	C250T	+	F	62	Parietal	Anaplastic	28	36	III	1	-	-	4.1
8	C228T	+	M	65	Sickle	Anaplastic	26	28	III	3	DP GI → GIII	-	A
9	C228T	+	F	70	Sickle	Anaplastic	20	31	III	2	DP GII → GIII	Lung	4.2
10	C250T	+	F	44	Frontal	Anaplastic	8	48	III	1	-	Lung	3.3
11	C228T	+	F	54	Sickle	Anaplastic	46	32	III	5	DP GI → GIII	-	2.6
12	C228T	+	M	48	Frontal	Anaplastic	22	7	III	2	DP GII → GIII	-	A
13	C250T	+	F	80	Frontal	Anaplastic	22	25	III	1	-	-	10

Abbreviations: ATRX, alpha-thalassemia/mental retardation syndrome X-linked; M, male; F, female; LI, labelling index; WHO, World Health Organization; OS, overall survival (months); NA, not available; A, alive; R, recurrence; DP, disease progression.

## Data Availability

Data sharing is not applicable to this article.

## Supplementary Materials

The following are available online at https://www.mdpi.com/article/10.3390/diagnostics11091624/s1, Table S1: Immunophenotypic and molecular profile of the cases selected for the study of the intra-tumour heterogeneity classified according to the WHO 2016 guidelines, Table S2: Phenotypic and genotypic features of the 25 selected tumour areas, Table S3: PCR and Sanger sequencing threshold as a function of genomic DNA quantification for the 25 selected tumour areas.
