# Supplementary material for: Human TERT Promoter Mutations in Atypical and Anaplastic Meningiomas
Source: Diagnostics (Basel). 2021 Sep 6;11(9):1624. doi: 10.3390/diagnostics11091624 (PMC8469948; doi:10.3390/diagnostics11091624)
Supplement: Supplementary file 1 [file diagnostics-11-01624-s001.zip › diagnostics-1327423-supplementary.pdf]

diagnostics

MDPI

Supplementary Table S1. Immunophenotypic and molecular profile of the cases selected for the study of the intra- tumour heterogeneity classified according to the WHO 2016 guidelines.

Case #	Morphologic variant	Brain parenchyma infiltration	Mitotic index	Ki-67 LI (%)	WHO grading < 2016	WHO grading > 2016	ATRX immunoreactivity	*pTERT* mutation status
14	Atypical	+	4	18.3	III	II	++	WT
15	Atypical	-	8	26.3	III	II	-	WT
16	Atypical	+	6	35.8	III	II	++	WT
17	Atypical	+	8	10	III	II	++	WT
18	Atypical	-	7	10.8	II	II	++	WT
19	Atypical	-	4	12.9	II	II	++	WT
20	Atypical	+	0	4.7	II	II	++	WT
21	Clear cell	+	< 4	17	II	II	++	WT
7	Anaplastic	+	28	36	III	III	++	C250T
22	Anaplastic	+	11	33.9	III	III	++	WT
23	Anaplastic	-	20	46.3	III	III	++	WT
24	Anaplastic	-	20	48.8	III	III	++	WT

Abbreviations: LI, labelling index; WHO, World Health Organization; ATRX, alpha-thalassemia/mental retardation syndrome X-linked; TERT, telomerase reverse transcriptase; WT, wild type.

Supplementary Table S2. Phenotypic and genotypic features of the 25 selected tumour areas.

Heterogeneity Criteria	Case #	Area	Dissection	Morphologic variant / WHO Grade	Brain Infiltration	Ki-67 LI (%)	ATRX Immunoreactivity
Morphologic Variant / WHO grade	**21**	**A**	**Macro**	**Clear cell**	**-**	**11.5**	++
		B	Macro	Papillary	-	4.9	++
		C	LMD	II	+*	13.17	+
	22	A	Macro	II	+	14.3	+
		B	Macro	III	-	33.9	++
	24	A	LMD	I	-	3.2	+
		B	Macro	III	-	48.8	++
Ki-67 LI	14	A	Macro	II	-	7.1	+
		B	Macro	II	-	18.5	+
	7	A	Macro	III	-	19.3	++
		B	Macro	III	-	36	++
	18	A	Macro	II	-	1.6	++
		B	LMD	II	-	7.2	++
	19	A	Macro	II	-	5.3	+
		B	LMD	II	-	9	++
	23	A	Macro	III	-	19	+
		B	Macro	III	-	46.3	++
Infiltration into brain parenchyma	15	A	LMD	II	-	26.3	-
		B	Macro	II	+	13	-
	16	A	Macro	II	-	25.8	+
		B	Macro	II	+	30	+
	17	A	Macro	II	-	2.8	+
		B	Micro	II	+	10	++
	20	A	Macro	II	-	2.7	+
		B	Macro	II	+	4.7	++

Abbreviations: WHO, World Health Organization; LI, labelling index; Macro, manual macrodissection; LMD, laser microdissection; ATRX, alpha-thalassemia/mental retardation syndrome X-linked. *Infiltration into brain parenchyma with perivascular cuffing.

Supplementary Table S3. PCR and Sanger sequencing threshold as function of genomic DNA quantification for the 25 selected tumour areas.

Heterogeneity criteria	Case #	Area	Surface (μm²)	Whitdrawals / thickness	Quantification			pTERT mutation status	
					DNA (ng/μl)	260/280	260/230	Primary tumour	Tumour area
Morphologic variant / WHO grade	21	A	Macro	13 (5-μm)	44.3	2.13	0.25	WT	WT
		B	Macro	13 (5-μm)	270.7	1.99	0.91		WT
		C	3,026,858	9	9.6	6.79	0.05		NA
	22	A	Macro	10 (5-μm)	210.9	1.94	0.85	WT	WT
		B	Macro	13 (5-μm)	384.2	1.94	1.15		WT
	24	A	5,104,412	12	9.5	4.27	0.06	WT	WT
		B	Macro	13 (5-μm)	818.7	1.87	1.45		WT
Ki-67 LI	14	A	Macro	15 (5-μm)	252.7	2	0.92	WT	WT
		B	Macro	13 (5-μm)	108.7	1.91	0.54		WT
	7	A	Macro	13 (5-μm)	494.6	1.91	1.28	C250T	C250T
		B	Macro	13 (5-μm)	100.4	1.97	0.49		WT
	18	A	Macro	10 (5-μm)	176.5	1.98	0.74	WT	WT
		B	3,845,611.5	9	10.4	4.94	0.06		WT
	19	A	Macro	20 (5-μm)	302.8	2	1.03	WT	WT
		B	4,634,689.2	18	8.4	47.7	0.05		WT
	23	A	Macro	10 (5-μm)	514.5	1.91	1.34	WT	WT
		B	Macro	13 (5-μm)	175.3	1.99	0.75		WT
Infiltration into brain parenchyma	15	A	4,397,022	12	10.3	3.93	0.05	WT	WT
		B	Macro	13 (5-μm)	92.5	1.94	0.45		C228T
	16	A	Macro	20 (5-μm)	153.3	2	0.66	WT	WT
		B	Macro	15 (5-μm)	124.1	2.02	0.56		WT
	17	A	Macro	10 (5-μm)	305.9	1.88	1.02	WT	WT
		B	2,555,109.7	9	8.1	9.87	0.05		WT
	20	A	Macro	10 (5-μm)	463.6	1.9	1.24	WT	WT
		B	Macro	10 (5-μm)	670.6	1.87	1.45		WT

Abbreviations: WHO, World Health Organization; LI, labelling index; Macro, manual macrodissection; LMD, laser micro-dissection; TERT, telomerase reverse transcriptase; WT, wild type.
